# Antibodies of IgG, IgA and IgM isotypes against cyclic citrullinated peptide precede the development of rheumatoid arthritis

**DOI:** 10.1186/ar3237

**Published:** 2011-02-03

**Authors:** Heidi Kokkonen, Mohammed Mullazehi, Ewa Berglin, Göran Hallmans, Göran Wadell, Johan Rönnelid, Solbritt Rantapää-Dahlqvist

**Affiliations:** 1 Departments of Public Health and Clinical Medicine/Rheumatology, Umeå University, SE-901 85 Umeå, Sweden; 2 Division of Clinical Immunology, Uppsala University, 751 85 Uppsala, Sweden; 3 Nutritional Research, Umeå University, SE-901 85 Umeå, Sweden; 4 Virology, Umeå University, SE-901 85 Umeå, Sweden

## Abstract

**Introduction:**

We and others have previously shown that antibodies against cyclic citrullinated proteins (anti-CCP) precede the development of rheumatoid arthritis (RA) and in a more recent study we reported that individuals who subsequently developed RA had increased concentrations of several cytokines and chemokines years before the onset of symptoms of joint disease. Here we aimed to evaluate the prevalence and predictive values of anti-CCP antibodies of IgG, IgM and IgA isotype in individuals who subsequently developed RA and also to relate these to cytokines and chemokines, smoking, genetic factors and radiographic score.

**Methods:**

A case-control study (1:4 ratio) was nested within the Medical Biobank and the Maternity cohorts of Northern Sweden. Patients with RA were identified from blood donors predating the onset of disease by years. Matched controls were selected randomly from the same registers. IgG, IgA and IgM anti-CCP2 antibodies were determined using EliA anti-CCP assay on ImmunoCAP 250 (Phadia AB, Uppsala, Sweden).

**Results:**

Of 86 patients with RA identified as blood donors prior to the onset of symptoms, samples were available from 71 for analyses. The median (Q1 to Q3) predating time was 2.5 years (1.1 to 5.9 years). The sensitivity of anti-CCP antibodies in the pre-patient samples was 35.2% for IgG, 23.9% for IgA, and 11.8% for IgM. The presence of IgG and IgA anti-CCP antibodies was highly significant compared with controls. IgG and IgA anti-CCP2 predicted RA significantly in conditional logistic regression models odds ratio (OR) = 94.1, 95% confidence interval (CI) 12.7 to 695.4 and OR = 11.1, 95% CI 4.4 to 28.1, respectively, the IgM anti-CCP showed borderline significance OR = 2.5 95% CI 0.9 to 6.3. Concentrations of all anti-CCP isotypes increased the closer to the onset of symptoms the samples were collected with an earlier and higher increase for IgG and IgA compared with IgM anti-CCP. IgA and IgG anti-CCP positive individuals had different patterns of up-regulated chemokines and also, smoking brought forward the appearance of IgA anti-CCP antibodies in pre-RA individuals.

**Conclusions:**

Anti-CCP2 antibodies of both the IgG and IgA isotypes pre-dated the onset of RA by years; also, both IgG and IgA anti-CCP2 antibodies predicted the development of RA, with the highest predictive value for IgG anti-CCP2 antibodies.

## Introduction

Rheumatoid arthritis (RA) is a chronic autoimmune disease characterized by joint inflammation involving the synovial tissue and ultimately leading to destruction of cartilage and bone. The pathogenic processes that lead to the development of the disease are not fully understood at this point.

We and others have shown that antibodies against citrullinated proteins/peptides (ACPA), analysed as anti-cyclic citrullinated proteins (CCP2) antibodies, precede the development of RA by several years [1,2] and that individuals who had the combination of anti-CCP antibodies together with either the human leukocyte antigen-shared epitope (HLA-SE) alleles or with the *protein tyrosine phosphatase non receptor type 22 (PTPN22) *1858T variant had a high relative risk of developing RA [3,4].

In a more recent study we also reported that individuals who subsequently developed RA had significantly increased levels of several cytokines and chemokines years before the onset of RA [5]. The pattern of the up-regulated cytokines, related factors and chemokines represented the adaptive immune system (that is, Th1, Th2 and Treg cell-related factors), while after disease onset the involvement and activation of the immune system appeared to be more general and wide-spread.

Currently little is known about the presence and prognostic significance of different isotypes of anti-CCP antibodies in RA. Studies have shown that anti-CCP2 antibodies of the IgG isotype are associated with radiographic progression in RA [6,7]. Investigators have shown that IgM anti-CCP2 antibodies are present in both early and established disease [8] and one later study showed that IgA and IgM anti-CCP2 antibodies were present in RA and were similarly specific for RA as IgG anti-CCP2 antibodies [9]. Patients with recent onset RA and positive for IgA anti-CCP2 antibodies were reported to suffer a more severe disease course over the first three years compared with patients negative for IgA anti-CCP2 antibodies [10] and the number of different isotypes has recently been related to the long-term radiographic progression in anti-CCP2 antibody positive RA patients [11].

In this study we aimed first to investigate the presence and predictive value of IgG, IgA and IgM isotypes of anti-CCP2 antibodies in individuals who subsequently developed RA and to assess their relation to rheumatoid factors (RFs) cytokines and chemokines, genetic factors, and smoking habits. Second, we evaluated the predictive effect of these predating antibodies for radiological progression after disease onset.

## Materials and methods

### Pre-patients and controls subjects

A nested case-control study designed with a 1:4 ratio was performed within the Medical Biobank of Northern Sweden and Northern Sweden maternity cohort. All individuals in the county of Västerbotten are continuously invited to donate to the Medical Biobank, the study cohort is population based and no one is excluded. All pregnant women screened for rubella constitute the maternity cohort. The conditions for recruitment and collection and storage of blood samples have been described previously [1] The register of patients with early RA attending the Department of Rheumatology at the University Hospital in Umeå and fulfilling the American College of Rheumatology classification criteria [12] for RA was co-analysed with those of the cohorts. The co-analyses identified 86 individuals (65 women and 21 men) who had donated blood samples before the onset of any symptoms of RA. Samples were available for analysis in this study from 71 of the 86 individuals identified. The median (interquartile range) period of time predating the onset of symptoms was 2.5 years (1.1 to 5.9 years). For every pre-patient, four control subjects were randomly selected from the same cohorts and matched for sex, age at the time of blood sampling, and area of residence. A total of 276 control subjects of 284 were available for analysis. Blood samples, collected at diagnosis of the disease, from 60 of the pre-patients were also available for analysis. The mean age of the patients at diagnosis was 54.3 years (range 27.9 to 68.3 years). Data on smoking history were collected and the donors were classified either as non-smokers or ever smokers (that is, either current and/or previous smokers). The mean time ± SD to diagnosis after the onset of symptoms was 7.0 ± 2.9 months. Anterior-posterior radiographs of the hands, wrists and feet obtained at baseline and after two years were graded according to the Larsen score [13].

This study was approved by the Regional Ethics Committee at the University Hospital, Umeå, Sweden, and all participants gave their written informed consent.

### Analyses of autoantibodies

#### IgG, IgA and IgM anti-CCP2 antibodies

IgG anti-CCP was determined using EliA anti-CCP assay on ImmunoCAP 250 (Phadia Diagnostic AB, Uppsala, Sweden), according to the manufacturer's instruction. All samples above the upper limit were diluted further in order to obtain precise values. This equipment uses wells coated with the CCP2 antigen, which has exactly the same composition as in other anti-CCP2 assays, that is, the Euro-Diagnostica ELISA assay (Arnhem, The Netherlands) used in our earlier report [1]. Most anti-CCP2 assays focus on quantitative measurements of autoantibody levels in the high (positive) range, when run according to the manufacturer's instructions, and do not always deliver quantitative results below the company-defined cut-off. The used equipment was originally developed for the measurement of specific IgE antibodies, that is, very low quantitative levels in the pg range. It also has a routine application for the measurements of conventional anti-CCP2 of the IgG isotype and also delivers quantitative values in the low range.

IgA anti-CCP was detected using the EliA IgA method on the ImmunoCAP 250. The method used the standard anti-CCP antigen solid phase from the same supplier as for the IgG anti-CCP measurements with an adaptation in the standard software. The method is used to detect specific human IgA antibodies against various antigens, by using an anti-human IgA detection antibody. All samples were diluted 1:100 prior to assay with samples above the upper limit being diluted further according to the manufacturer's instruction. The EliA IgA method is calibrated against WHO Ig reference preparation. Sample results were transformed from μg/l to U/ml according to assay lot specific correction factors. At the time of use no recommended cut off values were cited by the manufacturer.

IgM anti-CCP2 antibodies were analysed in the same way as IgA anti-CCP with a dilution of 1:100.

All analyses on the ImmunoCAP equipment were performed in Uppsala on aliquoted samples which had been stored at -70°C. In all preceding and actual procedures, patient and control samples were treated equally. Within-study reference ranges were defined by receiver operating characteristic (ROC) curves based on the studied population. Repeated analysis of individual samples during the study period showed stable values.

The anti-CCP antibody analyses used the same antigen as used in our earlier investigation of anti-CCP2 antibodies of the IgG isotype, and the IgG anti-CCP antibody analyses is consequently a reiteration of our earlier published anti-CCP2 data [1]. Rheumatoid factors of the IgA, IgG and IgM isotypes were determined using ELISAs, as previously described [1].

### Analysis of cytokines, cytokine receptors, and chemokines

The concentrations of 29 cytokines, cytokine related factors and chemokines were measured in plasma samples from 52 of the pre-patients, using multiplex detection kits from Bio-Rad (Hercules, CA, USA) as previously described [5]. The factors analysed were; Interleukin (IL)-1β, IL-2, IL-4, IL-5, IL-6, IL-7, IL-8, IL-9, IL-10, IL-12, IL-13, IL-15, IL-17, Eotaxin, IL-1 receptor antagonist (Ra), IL-2 receptor(R)alpha, basic fibroblast growth factor (FGF-basic), granulocyte colony stimulating factor (G-CSF), granulocyte-macrophage colony stimulating factor (GM-CSF), interferon (IFN)-γ, interferon-inducible protein (IP-10)/(CXCL10), monocyte chemo-attractant protein (MCP)-1/(CCL2), macrophage inflammatory protein (MIP)-1α/(CCL3), MIP-1β/(CCL4), platelet-derived growth factor-BB (PDGF-BB), tumor necrosis factor (TNF)-α, vascular endothelial growth factor (VEGF), monokine induced by interferon-γ (MIG/CXCL9), and macrophage-migration inhibitory factor (MIF).

### Analysis of genetic factors

HLA-DRB1 genotyping for 0404 and 0401 was performed using polymerase chain reaction (PCR) sequence-specific primers from an HLA-DR low-resolution kit and a HLA-DRB1*04 sub-typing kit as previously described [3]. The *PTPN22 *1858C/T polymorphism (rs2476601) was determined using an ABI PRISM 7900HT Sequence Detector System (Applied Biosystems, Foster City, CA, USA) as previously described [4].

### Statistical analysis

Statistical calculations were performed using SPSS for Windows version 17.0 (SPSS; Chicago, IL, USA). Continuous data were compared by non-parametric analyses with Wilcoxon's signed rank test for matched pairs (pre-patients versus RA patients) and conditional logistic regression analyses (pre-patients versus matched controls).

Relationships between categorical data (positive versus negative) were compared using Chi-square analyses or Fisher's exact test, when appropriate. Continuous data within the patient group were assessed using the Student's *t*-test for independent samples when appropriate. Variations over time of continuous data within and between groups were assessed with the Kruskal-Wallis test or with paired samples t-test (pre-patients versus RA patients). *P*-value < 0.05 was considered statistically significant. No correction for the number of comparisons was made unless explicitly stated so. Receiver operating characteristic (ROC) curves were constructed for the three anti-CCP2 antibody isotypes to identify cut-offs according to the value resulting in the combination of the highest sensitivity and specificity based on samples from the patients together with the controls. The cut-off for IgG anti-CCP was 15 U/ml, for IgA anti-CCP 2.5 U/ml and for IgM anti-CCP 90 U/ml.

## Results

Characteristics of the individuals with samples before disease onset (pre-patients) and population based matched controls are presented in Table 1.

**Table 1:** Characteristics of the pre-patients (individuals before the onset of any symptoms of joint disease) and matched controls

	Pre-patients	Controls
Number (% female)	71 (80.3)	276 (80.1)
Age (range), years	48.6 (19.6 to 66.9)	50.0 (18.6 to 69.1)
Smoking ever, n (%)	49 (69.0)***	122/271 (45.0)
HLA-SE alleles, n (%)	38/68 (55.9)**	39/116 (33.6)
*PTPN22 *1858T, n (%)	28 (39.4)**	47/210 (22.4)
Anti-CCP2 antibodies^E ^(%)	23 (32.4)***	4 (1.5)
IgM RF, n (%)	17 (22.4)***	14 (5.1)
IgA RF, n (%)	28 (36.8)***	12 (4.3)
IgG RF, n (%)	15 (19.7)***	14 (5.1)

**P < 0.01, ***P < 0.001, ^E ^analysed with a kit from Euro-Diagnostica. HLA-SE, human-leukocyte antigen - shared epitope; *PTPN22*, protein tyrosine phosphatase non-receptor type 22; Anti-CCP, anti-cyclic citrullinated peptide; Ig, immunoglobulin; RF, rheumatoid factor.

Samples from the 71 individuals before they presented with any symptoms of joint disease and 276 matched controls analysed for the presence of anti-CCP2 antibodies of the IgG, IgA and IgM isotypes showed significantly increased levels, and the concentrations were further increased when these individuals were diagnosed with RA (Table 2). The sensitivity for the different isotypes was in the pre-patients, 35.2% for the IgG, 23.9% for IgA, and 11.8% for IgM with a specificity of 98.9%, 97.1%, and 93.9%, respectively (Table 3). The sensitivity for IgG and IgA anti-CCP were highly significant in samples from pre-RA patients compared with controls, whereas IgM anti-CCP did not reach statistical significance (Table 3). The sensitivity for all anti-CCP2 antibody isotypes were highly significant in samples when patients were diagnosed with RA compared with controls (Table 3).

**Table 2:** Concentrations (U/ml) (median values (IQR)) of anti-CCP2 antibodies in pre-patients, matched controls and RA patients

	IgA-CCP2	IgG-CCP2	IgM-CCP2
Controls (*n *= 276)^a^	0.8	1.2	18.5
(IQR)	(0.5 to 1.1)	(0.8 to 2.1)	(11.1 to 34.3)
Pre-patients (*n *= 71)^b^	1.1*	3.4 **	21.6 *
(IQR)	(0.6 to 2.1)	(1.2 to 55.0)	(10.1 to 55.3)
Patients (*n *= 53)^c^	1.8***	14.0 ***	51.9***
(IQR)	(0.9 to 6.8)	(11.4 to 715.5)	(29.8 to 127.0)
p value^1^	<0.001	<0.001	<0.001

^a ^Analysis of IgM; *n *= 263 controls ^b^, Analysis of IgM; *n *= 68 pre-patient samples, ^c^Analysis of IgA; *n *= 54 and of IgG-CCP2; *n *= 59, * *P *< 0.05, ** *P *< 0.01, ***P < 0.001 pre-patients vs. controls or RA patients vs. controls,^1^*P*-values obtained from Kruskal-Wallis analysis comparing all three groups. Anti-CCP, anti-cyclic citrullinated peptide; IQR, inter quartile range; Ig, immunoglobulin.

**Table 3:** Sensitivity and specificity for various anti-CCP2 antibody isotypes for 71 pre-patients, 274 matched controls and 60 patients

Anti-CCP antibody	Sensitivity	Sensitivity	Specificity
	pre-patients	patients	
IgG^P^	33.8***	71.7***	98.9
IgA^P^	23.9***	43.3***	97.1
IgM^aP^	11.8	32.8***	93.9
IgG^E^	32.4***	71.2***	98.5

The cut-off values for positivity for the antibodies were calculated using ROC curves

^a^Analysis of IgM in pre-patients; *n *= 68, in patients; *n *= 58, and controls; *n *= 263, ^E ^analysed with a kit from Euro-Diagnostica, ^P ^analysed with a kit from Phadia.***P < 0.001. Anti-CCP, anti-cyclic citrullinated peptide; ROC, receiver operating characteristic; Ig, immunoglobulin.

The predictive value of various factors for developing RA analysed in simple conditional logistic regression was highest for the IgG anti-CCP2 test followed by IgA anti-CCP. Smoking had a higher odds ratio for developing RA than HLA-SE, carriage of the *PTPN22 *1858T variant and IgM anti-CCP (Table 4). In multiple conditional regression analyses adjusted for HLA-SE, *PTPN22 *1858T variant and smoking, respectively, only the odds ratio for IgM anti-CCP increased to 3.89, 95% CI 1.36 to 11.10, *P *< 0.05 when adjusted for the *PTPN22 *1858T variant. In multiple conditional logistic regression analyses including IgG and IgA anti-CCP antibodies the only isotype that remained significant was the IgG isotype, *P *< 5.9 × 10^-5 ^(data not shown).

**Table 4:** Simple conditional logistic regression analyses of the different isotypes of anti-CCP2 antibodies, RFs, HLA-SE, *PTPN22 *1858T and smoking in pre-patients and matched controls

Factors	OR (95%CI)	*P*-value
IgG-CCP2	94.11 (12.74 to 695.41)	<0.0001
IgA-CCP2	11.07 (4.36 to 28.09)	<0.0001
IgM-CCP2	2.49 (0.98 to 6.29)	0.054
IgG RF	7.01 (2.82 to 17.42)	<0.0001
IgA RF	15.87 (6.53 to 38.58)	<0.0001
IgM RF	6.56 (2.77 to 15.50)	<0.0001
HLA-SE	2.91 (1.47 to 5.76)	0.002
*PTPN22 *1858T	2.18 (1.15 to 4.12)	0.017
Smoking	3.12 (1.73 to 5.63)	<0.0001

Only one sample from each individual has been included in the analyses.

Anti-CCP, anti-cyclic citrullinated peptide; HLA-SE, human-leukocyte antigen - shared epitope; *PTPN22*, protein tyrosine phosphatase non-receptor type 22; Ig, immunoglobulin.

**Figure 1:**
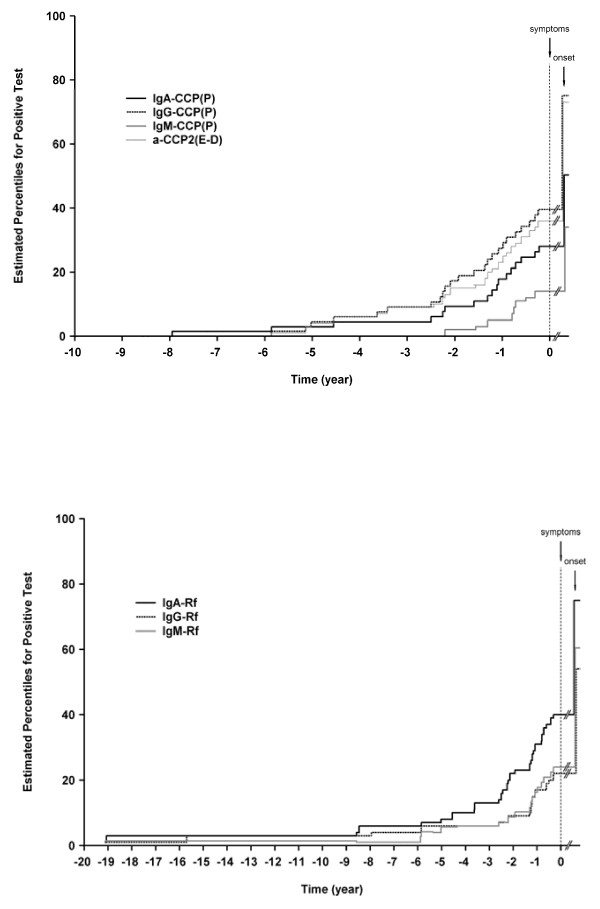
**Accumulated percentage positive samples of IgA, IgG and IgM isotypes**. **(a) **Accumulated percentage positive samples of IgA, IgG and IgM anti-CCP2 isotypes analysed using Phadia (P) and IgG anti-CCP2 using Euro-Diagnostics (E-D) in individuals before onset of symptoms and at diagnosis of RA. **(b) **Accumulated percentage positive samples of IgA, IgG and IgM isotypes antibodies of rheumatoid factor in individuals before onset of symptoms and at diagnosis of RA.

The accumulated percentage of positive samples (above cut-off according to ROC-curves) for each analysis is presented in Figure 1a. The accumulated positivity for IgG anti-CCP2 test was in almost complete concordance with the previously analysed IgG anti-CCP2 test using Euro-Diagnostica [1] while the positivity for both IgA and IgM anti-CCP were lower (Figure 1a). Positivity for IgA was apparent before that of IgM and was higher during all time points preceding the onset of symptoms. This pattern for the three isotypes was quite different from that for RF isotypes where the first isotype to appear was IgA followed by IgG and IgM; the two latter were almost in complete concordance until less than 0.5 months before disease onset (Figure 1b). In pre-patients the presence of anti-CCP antibodies, IgG and IgA isotypes, was significantly associated with RA, irrespective of positivity for IgM, IgG or IgA-RFs (data not shown). IgM-RF was independent of IgA anti-CCP antibody isotype associated with RA but not of IgG anti-CCP isotype in pre-patients. In the RA patients both anti-CCP antibodies (IgG and IgA isotypes) and IgM-RF were independently associated with RA.

In a multiple logistic regression analysis including IgM-RF and IgG anti-CCP isotype the relative risk for development of RA was not significant for IgM-RF alone, but the odds ratio for IgG anti-CCP isotype was 32.4 (9.0 to 116.9), P < 10^-6^. The odds ratio further increased to 71.2 (9.9 to 566.7), P < 10^-4 ^by combination of IgM-RF and IgG isotype suggesting an interaction of IgM-RF with IgG anti-CCP antibody isotype in pre-patients. In similar analysis of IgM-RF and IgA isotype of anti-CCP antibodies the combination yielded a higher odds ratio ((38.7 (4.6 to 321.6), *P *< 0.001) than for the individual antibodies alone (odds ratio 3.8 (1.5 to 9.5), *P *< 0.01 and 6.9 (2.6 to 18.4), *P *< 0.001, respectively).

**Figure 2:**
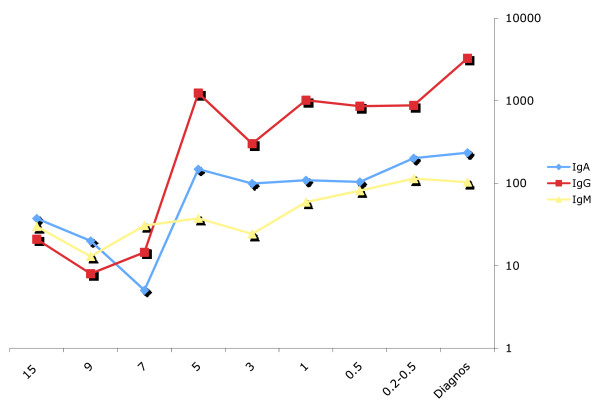
**Concentrations, in percentage of cut off value of anti-CCP2 antibody isotypes before and at disease onset**.

The concentration of IgG anti-CCP2 antibodies analysed in comparison for every separate individual increased significantly over time and until the onset of RA (*P *< 0.0001). The increase was already detectable in samples from individuals collected up to five years before onset of symptoms and thereafter the concentrations remained fairly constant until 0.25 years before onset of symptoms. Individuals sampled at the time point of diagnosis compared with those 0.25 years before onset of symptoms had significantly higher concentrations of IgG anti-CCP (*P *< 0.05; Figure 2). There was also a significant gradual increase in the concentration of IgA anti-CCP until 0.25 years before onset of symptoms (*P *< 0.05, including all time points before onset of symptoms) and, analyzing all time points including data at diagnosis (*P *< 0.01). There was no significant increase in concentrations of IgA anti-CCP just before and when the disease was diagnosed in contrast to the increase in concentration of IgG anti-CCP (Figure 2). There was also a significant increase in the concentration of the IgM anti-CCP over time until 0.25 years before onset of symptoms (*P *< 0.02) and at diagnosis (*P *< 0.001, including all time points) although it was evident that the increase started later than for the other isotypes; that is, actually three years or less before onset of symptoms (Figure 2).

In pre-patients stratified for the absence or presence of the different anti-CCP2 isotypes the pattern of significantly increased cytokines was rather similar in IgA and IgG anti-CCP2 antibody positive individuals with increased concentrations of cytokines involved in general immune activation such as IL-2 and IL-6, the Th1 related cytokine IL-12, several Th2 related cytokines (IL-4, IL-9 and Eotaxin) and VEGF compared with antibody negatives (Table 5). In IgA anti-CCP2-positive individuals there was also significantly increased concentrations of IL-1β and GM-CSF, whereas in IgG anti-CCP2 antibody positive individuals the concentrations of IL-15, IFN-γ and IL-17 was significantly increased (Table 5). However, after correction for multiple comparisons only IL-2Rα and IL-2 in IgG isotype positive individuals remained significantly increased *P *< 0.05 and *P *< 0.01, respectively). There was a difference in the patterns of chemokines related to the different anti-CCP isotypes, where the only chemokine significantly increased in IgG anti-CCP2-positive individuals was MIG, whereas in IgA positive individuals the concentrations of MCP-1, MIP-1β, IP-10 and MIG were all significantly elevated compared with IgA negative individuals (Table 5). MIG was the only chemokine remaining significantly increased after correction for multiple comparisons. There were no significant differences in the concentrations of cytokines and chemokines between IgM anti-CCP2 positive and negative pre-RA patients (data not shown).

**Table 5:** Concentrations of cytokines, cytokine related factors and chemokines (median, IQR, pg/mL) in pre-patients stratified for the presence or absence of IgA and IgG isotypes of anti-CCP2 antibodies

	IgA positive	IgA negative	*P*-value	IgG positive	IgG negative	*P*-value
	(*n *= 14)	(*n *= 38)		(*n *= 20)	(*n *= 32)	
Cytokine/chemokine						
*General activation*						
IL-1β	7.2 (2.9 to 36.4)	3.4 (2.3 to 5.1)	**0.025**	5.9 (2.3 to 25.5)	3.3 (2.3 to 4.9)	0.071
IL-1Ra	164.6 (128.7 to 454.9)	144.1 (96.5 to 195.5)	0.089	162.9 (109.7 to 408.6)	144.1 (99.8 to 186.5)	0.136
IL-2Rα	66.9 (38.6 to 110.0)	33.4 (22.3 to 51.3)	**0.008**	60.9 (35.9 to 88.7)	32.2 (20.8 to 47.6)	**<0.001**
TNFα	108.1 (3.0 to 506.7)	49.1 (25.7 to 118.6)	0.556	85.4 (27.2 to 224.0)	45.0 (20.8 to 112.9)	0.234
IL-6	19.8 (5.9 to 147.7)	4.3 (1.1 to 11.5)	**0.009**	16.2 (3.6 to 111.6)	4.8 (1.1 to 8.2)	**0.019**
IL-2	74.7 (2.8 to 141.5)	3.8 (1.1 to 18.3)	**0.020**	49.1 (10.5 to 106.8)	1.4 (1.1 to 11.7)	**<0.001**
IL-15	2.4 (0.2 to 9.6)	0.5 (0.2 to 4.2)	0.355	4.2 (0.8 to 4.2)	0.5 (0.2 to 4.2)	**0.010**
*Th1 related*						
IL-12	54.4 (21.0 to 135.2)	21.2 (13.7 to 32.3)	**0.033**	53.5 (21.4 to 161.3)	20.5 (13.5 to 29.3)	**0.012**
IFN-γ	113.4 (84.5 to 1711.0)	92.9 (50.5 to 150.1)	0.083	152.2 (97.1 to 1167.6)	78.5 (50.4 to 130.8)	**0.022**
*Th2 related*						
IL-4	4.4 (3.2 to 26.4)	3.0 (2.2 to 4.2)	**0.044**	4.4 (3.3 to 17.4)	2.9 (2.3 to 4.0)	**0.019**
IL-5	5.6 (1.3 to 12.0)	5.8 (3.3 to 7.7)	0.675	5.8 (1.3 to 12.0)	5.8 (3.6 to 7.3)	0.441
IL-9	128.3 (22.7 to 283.6)	19.6 (5.7 to 85.9)	**0.022**	121.1 (24.7 to 255.9)	17.6 (5.6 to 71.7)	**0.012**
IL-13	5.1 (3.6 to 11.2)	4.4 (2.9 to 6.7)	0.329	5.3 (3.9 to 17.0)	4.0 (3.0 to 6.5)	0.089
Eotaxin	80.7 (43.2 to 429.8)	42.0 (26.1 to 53.1)	**0.008**	80.7 (44.0 to 363.8)	36.7 (26.0 to 47.6)	**0.004**
*Th17 related*						
IL-17	31.3 (17.7 to 46.5)	22.3 (9.9 to 38.1)	0.082	31.5 (20.3 to 42.0)	21.2 (9.6 to 36.9)	**0.040**
*Treg cell-related*						
IL-10	6.5 (3.8 to 19.8)	5.5 (3.6 to 8.1)	0.279	6.2 (3.2 to 18.4)	5.3 (3.9 to 7.6)	0.541
*Bone marrow derived*						
IL-7	23.8 (19.2 to 36.8)	27.2 (18.6 to 37.4)	0.807	24.8 (13.4 to 42.1)	26.2 (20.8 to 36.6)	0.620
GM-CSF	23.0 (12.9 to 103.4)	5.0 (2.5 to 23.9)	**0.008**	20.2 (5.5 to 62.0)	4.6 (2.5 to 15.7)	0.054
G-CSF	57.1 (39.0 to 80.1)	58.7 (42.3 to 74.5)	0.463	57.1 (38.8 to 83.1)	58.7 (43.6 to 72.7)	0.527
*Stromal cells and angiogenic factors*						
bFGF	6.7 (2.2 to 14.1)	6.6 (2.2 to 6.8)	0.187	2.9 (1.6 to 11.2)	6.6 (3.5 to 6.8)	0.519
PDGF-BB	1905.9 (705.9 to 2387.4)	1985.6 (997.6 to 3160.0)	0.911	1975.9 (1109.6 to 3437.3)	1695.3 (950.6 to 2370.9)	0.312
VEGF	24.6 (10.0 to 63.7)	13.1 (5.7 to 23.9)	**0.029**	28.6 (12.0 to 53.1)	11.7 (4.9 to 15.0)	**0.022**
*Chemokines*						
MIF	367.1 (90.8 to 538.2)	223.0 (142.1 to 461.5)	0.919	384.5 (163.2 to 599.4)	207.8 (123.7 to 366.5)	0.095
MIG	488.5 (319.0 to 1167.5)	272.9 (190.4 to 371.2)	**0.004**	425.6 (279.1 to 741.3)	272.9 (201.6 to 364.0)	**0.039**
IL-8	7.7 (3.6 to 11.4)	5.0 (0.5 to 10.2)	0.263	6.4 (0.5 to 12.1)	6.6 (1.7 to 9.4)	0.975
IP-10	962.1 (696.2 to 2214.2)	634.3 8425.0 to 1062.0)	**0.014**	957.0 (644.2 to 1855.8)	624.4 (420.0 to 1071.5)	0.061
MCP-1	23.0 (19.0 to 62.4)	18.5 (12.8 to 28.4)	**0.028**	25.8 (17.6 to 52.7)	17.3 (12.9 to 26.3)	0.085
MIP-1α	7.2 (4.2 to 11.7)	6.9 (4.8 to 10.0)	0.746	7.4 (2.9 to 11.9)	6.9 (5.1 to 9.3)	0.994
MIP-1β	44.2 (37.6 to 49.7)	35.7 (26.5 to 42.2)	**0.030**	42.9 (31.4 to 48.7)	36.5 (27.4 to 41.1)	0.200

IQR, inter quartile range; Ig, immunoglobulin; anti-CCP, anti-cyclic citrullinated peptide; IL, interleukin; TNF, tumor necrosis factor; IFN; interferon, GM-CSF, granulocyte macrophage colony stimulating factor; G-CSF, granulocyte colony stimulating factor; bFGF, basic-fibroblast growth factor; PDGF, platelet derived growth factor; VEGF, vascular endothelial growth factor; MIF, macrophage-migration inhibitory factor; MIG, monokine induced by interferon; MCP, monocyte chemoattractant protein; MIP, macrophage inflammatory protein.

Pre-patient smokers had increased risk for developing IgG anti-CCP (*P *= 0.04), while there was no relationship between smoking and the other anti-CCP isotypes. Although, in pre-RA patients who were smokers, IgA anti-CCP antibodies appeared significantly earlier than in non-smokers. The mean time was 2.4 years before the onset of symptoms in smokers compared with 0.6 years in non-smokers. (*P *= 0.01). There was no difference for IgG and IgM anti-CCP in smokers versus non-smokers. Also pre-RA patients who were smokers were significantly more often IgA RF positive (*P *= 0.02). There were no differences for the other RF isotypes related to smoking habits (data not shown).

The predictive effect on radiological destruction at disease onset and after 24 months of the different anti-CCP2 isotypes in pre-patients was evaluated. The Larsen score (mean ± SEM) at baseline (when diagnosed with RA) was significantly higher in patients with IgG (6.6 ± 1.4) and IgM (9.2 ± 1.6) anti-CCP2 versus patients negative for the corresponding antibodies (3.4 ± 0.7 and 3.8 ± 0.7, respectively) for these isotypes before symptoms of joint disease (*P *< 0.05 for both isotypes). Twenty-four months after diagnosis the Larsen score was significantly higher in patients positive for IgG and IgA anti-CCP (13.4 ± 2.4 and 13.6 ± 3.7, respectively) but not for IgM before symptoms of disease compared with those negative for the corresponding antibody. There were also significant differences in Larsen score at baseline between individuals positive for 0, 1, 2 or more anti-CCP isotypes before the onset of symptoms (mean score 2.9, 4.9 and 8.2, respectively, *P *< 0.05). The same was observed for Larsen score after 24 months (mean score 6.9, 8.5, and 17.8 respectively, *P *< 0.05). Overall there was a significant increase in Larsen score in all subgroups after 24 months compared with baseline.

Individuals carrying HLA-SE alleles also had to a greater extent IgG anti-CCP2 antibodies than those lacking these alleles, although the difference was not significant (*P *= 0.070). None of the other isotypes of anti-CCP2 was associated with the carriage of HLA-SE alleles. There was no relationship between any of the anti-CCP2 antibodies and the *PTPN22 *1858T variant (data not shown).

## Discussion

In this study, we analysed the concentrations of the IgG, IgA and IgM isotypes of anti-CCP2 antibodies and constructed ROC curves for the studied population. Furthermore, we also related the presence of antibodies of each isotype with previously analysed IgG, IgA and IgM RFs, cytokines, cytokine related factors and chemokines collected at the same time points. In samples from individuals before onset of joint symptoms, IgG and IgA anti-CCP, with a higher frequency of individuals positive for IgG anti-CCP, were the first antibodies to appear before disease onset. IgM anti-CCP2 appeared later and with a lower frequency. The anti-CCP isotype pattern was quite different compared with RF. Sporadic cases positive for all RF isotypes appeared much earlier in time with the highest accumulated frequency of positive individuals for IgA RF. IgG and IgM RF were very similar until less than 0.25 years before onset. Then just before the onset the frequency of IgA and IgM RFs markedly increased. To our knowledge, this is the first study analyzing different isotypes of anti-CCP2 antibodies before onset of symptoms of disease, thus there are no comparable data. However, there are data published on patients with established RA showing the same pattern for anti-CCP2 isotypes with the highest frequency of IgG (74.8%), followed by IgA (52.9%) and IgM (44.5%) [9]. Also, it was shown that antibodies against anti-viral citrullinated peptide of IgG and IgA isotypes had a high specificity for discriminating RA [14]. In line with Verpoort *et al.*, the frequency of individuals positive for IgA anti-CCP2 was higher in RA than in undifferentiated arthritis (UA) [8]. However, in our study of individuals before onset of RA no limitations of included subjects were performed compared with Verpoort *et al.*, where only IgG anti-CCP positive subjects were included [8]. In comparing the contribution from the separate antibodies, we found that IgG and IgA were associated with RA independent of IgM-RF both in pre-patients and RA patients. IgM-RF was dependent of IgG anti-CCP isotype in predicting RA in pre-patients but not in RA patients. The analyses (both of IgG and of IgA anti-CCP isotypes, respectively) and IgM-RF in combination indicated an increase in odds ratio for developing RA compared with having each antibody separately, which is in line with the findings by Ioan-Facsinay *et al. *[15].

In this paper the diagnostically most important anti-CCP isotype was IgG, followed by IgA and IgM in pre-patients. This order differed totally from our earlier published RF isotype data, where IgA RF had the strongest diagnostic impact, followed by IgM and then IgG RF [1]. This inconsistency partly reflects the routine clinical use of these antibodies where IgG anti-CCP and IgM (and sometimes IgA) RF have the major diagnostic strength; whereas IgG RF has a very low diagnostic sensitivity. Hypothetically the IgM-dominated RF response might represent a non-isotype switched T cell independent immune response whereas the IgG-dominated CCP pattern might reflect a mature T cell dependent immune reaction. The pattern is, however, complex, as the IgM anti-CCP increase appeared late, just a few years before onset, in pre-patient samples, as could be expected in a normal T dependent immune response, and as IgA RF, representing a more mature isotype, appeared before IgM RF.

As this study on ACPA in pre-RA samples concerns the gradual increase from within the normal range to highly positive levels and most ELISA based anti-CCP assays focus on measurement of autoantibody levels in the high (positive) range, we decided to instead apply a fluorescence-based assay to be able to deliver quantitative levels also in the normal range. By adjusting this equipment that routinely measures IgG anti-CCP we were able to measure also IgA and IgM anti-CCP levels in pre-RA and control samples.

In our previous paper we found higher levels of cytokines and chemokines in IgG anti-CCP positive patients, [5] which was in line with the publication by Hueber *et al. *[16]. Therefore, we undertook analyses of the cytokine and chemokine concentrations stratified for the different anti-CCP2 isotypes. In both IgA and IgG anti-CCP2 positive individuals there was a similar pattern with increased levels of proinflammatory cytokines (IL-2 and IL-6), related factor (IL-2Ra), Th1 related cytokine (IL-12), Th2 related cytokines (IL-4, eotaxin and IL-9), and VEGF. On the other hand, there was a striking difference in pre-RA patients between individuals with IgA and IgG anti-CCP2 antibodies, where the levels of a number of chemokines (IP-10, MCP-1 and MIP-1ß) were significantly increased only in IgA anti-CCP positive individuals. The only chemokine remaining significant after correction for multiple comparisons was MIG in IgA anti-CCP positives. We are not sure how to interpret these results. In one study a patient group were described to cluster together in microarray analysis having lower levels of IFN-γ and TNF but high expression of MCP-1 and MIP-1ß and was unlikely to associate with anti-CCP2 antibodies (that is, of the IgG isotype) compared with other groups related to anti-CCP2 antibodies [17]. These findings are interesting in the light of our findings, that is, individuals with positivity for IgA isotype who did not have increased concentrations of TNF-and IFN-γ but of MCP-1 and MIP-1ß. Of the IgA anti-CCP positive individuals analysed for cytokines and chemokines, 78.6% were also IgG positive in this study. Given our limited cohort size it is difficult to separate the effects of IgG and IgA anti-CCP, and these results should optimally be repeated in a much larger pre-patient cohort.

In two studies it was shown that smokers had a higher frequency of IgA [10,18] and IgM [18] anti-CCP2 antibodies than patients who were non-smokers. In our study there was an association between smoking and the development of IgG anti-CCP2 antibodies and IgA RF pre-diagnosis but not for the other autoantibody isotypes, although the analysed subgroups were small. However, in smokers IgA anti-CCP2 antibodies appeared with a significantly longer predating time compared with non-smokers positive for the IgA isotype. This could indicate that smoking could be an environmental trigger for the appearance not only of ACPA in general but especially for the IgA isotype before onset of disease. The association between smoking and IgA was not limited to anti-CCP antibodies. Our finding that smokers were IgA RF-positive significantly more often than non-smokers among individuals before onset of symptoms of disease implies that smoking pre-diagnosis might have a connection with a humoral autoimmunity switch to IgA production. The subgroups in this study are, however, small, and the results have to be interpreted with caution.

Studies have shown that the presence of anti-CCP antibodies is related to the radiographic progression in RA [6,7] and the combination of IgA and IgG isotypes have been suggested to identify a group of more severely affected RA patients [10]. Recently a publication by van der Woude *et al. *showed that in IgG anti-CCP2 antibody positive patients, the presence of more anti-CCP antibody isotypes at baseline was associated with higher radiographic score [11]. In our study we extend this by showing that patients positive for more anti-CCP2 isotypes already before onset of symptoms had a higher radiographic score both at baseline and after 24 months of disease compared with pre-RA individuals with fewer or no anti-CCP2 isotypes before onset.

There are limitations to this study, such as the low number of samples available, which makes it difficult to stratify into subgroups. The effects of sample storage time in small aliquots are also to be considered. This was, however, compensated for by selecting controls that were matched for the date of sampling and storage conditions. The results of our analysis of IgG anti-CCP2 antibodies previously published [1] performed years before the present IgG anti-CCP2 analysis was in almost complete agreement with the present results, also strongly arguing for the stability of the samples.

## Conclusions

Anti-CCP2 antibodies of both the IgG and IgA isotypes pre-dated the onset of RA by several years and also, antibodies of both IgG and IgA isotypes predicted the development of RA, with the highest predictive value for IgG anti-CCP2 antibodies. There was a difference in the pattern of up-regulated chemokines in IgA and IgG anti-CCP positive individuals, and smoking brought forward the appearance of IgA anti-CCP2 pre-RA; findings that could indicate that these isotypes have different functions in the pathogenesis of RA.

## Abbreviations

ACPA: antibodies against citrullinated proteins/peptides; anti-CCP: anti-cyclic citrullinated peptide; ELISA: enzyme linked immunosorbent assay; basic-FG: fibroblast growth factor; CI: confidence interval; G-CSF: granulocyte colony stimulating factor; GM-CSF: granulocyte macrophage colony stimulating factor; HLA: human leukocyte antigen; IL: interleukin; IP-10: interferon inducible protein; IQR: interquartile range; MCP-1: monocyte chemo-attractant protein; MIF: macrophage-migration inhibitory factor; MIG: monokine induced by interferon; MIP-1: macrophage inflammatory protein; OR: odds ratio; PCR: polymerase chain reaction; PDGF-BB: platelet derived growth factor - BB; PTPN22: protein tyrosine phosphatase non receptor type 22; RA: rheumatoid arthritis; RF: rheumatoid factor; ROC curve: receiver operating characteristic curve; SD: standard deviation; SE: shared epitope; SEM: standard error mean; VEGF: vascular endothelial growth factor.

## Competing interests

The authors declare that they have no competing interests.

## Authors' contributions

HK, the main investigator, performed the analysis of cytokines, carried out the statistics and contributed to preparation of the manuscript. MM established the IgA and IgM anti-CCP analyses, and carried out and interpreted all anti-CCP antibody analyses on the Phadia platform. EB participated in the radiological scoring of the patients. GH and GW are responsible for the Medical Biobank and Maternity cohort, respectively. JR participated in the design of the study, was responsible for the anti-CCP antibody analyses and participated in finalizing the manuscript. SRD, the principal investigator, was responsible for the Biobank samples, designed the investigation and participated in data collection, statistical analysis and drafting of the manuscript. All authors have read and approved the final manuscript.
